# Structuring Detergents for Extracting and Stabilizing Functional Membrane Proteins

**DOI:** 10.1371/journal.pone.0018036

**Published:** 2011-03-31

**Authors:** Rima Matar-Merheb, Moez Rhimi, Antoine Leydier, Frédéric Huché, Carmen Galián, Elodie Desuzinges-Mandon, Damien Ficheux, David Flot, Nushin Aghajari, Richard Kahn, Attilio Di Pietro, Jean-Michel Jault, Anthony W. Coleman, Pierre Falson

**Affiliations:** 1 CNRS, Drug Resistance Mechanism and Modulation Laboratory, Ligue labeled team 2009, Lyon, France; 2 Laboratoire de BioCristallographie et Biologie Structurale des Cibles Thérapeutiques, Université Lyon 1, Univ Lyon; CNRS, UMR 5086; Bases Moléculaires et Structurales des Systèmes Infectieux, IBCP, Lyon, France; 3 CEA DEN/DRCP/SCPS/LEPS, Bagnols-sur-Cèze, France; 4 CNRS/CEA/Université Joseph Fourier, Institut de Biologie Structurale, Grenoble, France; 5 Laboratoire des Multimatériaux et Interfaces, UMR 5615 CNRS/Université de Lyon 1, Lyon, France; 6 European Synchrotron Radiation Facility, BP 220, Grenoble, France; Deutsches Krebsforschungszentrum, Germany

## Abstract

**Background:**

Membrane proteins are privileged pharmaceutical targets for which the development of structure-based drug design is challenging. One underlying reason is the fact that detergents do not stabilize membrane domains as efficiently as natural lipids in membranes, often leading to a partial to complete loss of activity/stability during protein extraction and purification and preventing crystallization in an active conformation.

**Methodology/Principal Findings:**

Anionic calix[4]arene based detergents (C4Cn, n = 1–12) were designed to structure the membrane domains through hydrophobic interactions and a network of salt bridges with the basic residues found at the cytosol-membrane interface of membrane proteins. These compounds behave as surfactants, forming micelles of 5–24 nm, with the critical micellar concentration (CMC) being as expected sensitive to pH ranging from 0.05 to 1.5 mM. Both by ^1^H NMR titration and Surface Tension titration experiments, the interaction of these molecules with the basic amino acids was confirmed. They extract membrane proteins from different origins behaving as mild detergents, leading to partial extraction in some cases. They also retain protein functionality, as shown for BmrA (*Bacillus m*ultidrug *r*esistance *A*TP protein), a membrane multidrug-transporting ATPase, which is particularly sensitive to detergent extraction. These new detergents allow BmrA to bind daunorubicin with a Kd of 12 µM, a value similar to that observed after purification using dodecyl maltoside (DDM). They preserve the ATPase activity of BmrA (which resets the protein to its initial state after drug efflux) much more efficiently than SDS (sodium dodecyl sulphate), FC12 (Foscholine 12) or DDM. They also maintain in a functional state the C4Cn-extracted protein upon detergent exchange with FC12. Finally, they promote 3D-crystallization of the membrane protein.

**Conclusion/Significance:**

These compounds seem promising to extract in a functional state membrane proteins obeying the positive inside rule. In that context, they may contribute to the membrane protein crystallization field.

## Introduction

Membrane proteins represent 20–30% of total proteins in all species [1] and up to 60% of pharmaceutical targets [2]. However, as of today, only 1% have been structurally characterized [3], seriously compromising structure-based drug design. Several difficulties hinder structural studies of membrane proteins [4]. They are generally not abundantly expressed in cells and are refractory to overexpression, especially for eukaryotic proteins [5]. The major bottleneck for their crystallization arises from their instability outside a lipid bilayer environment. To be purified, they first need to be extracted by detergents, forming complex micelles where looser packing can lead to unfolding and loss of activity [6]. Micelle-forming detergents are also well known for their ability to reduce protein-protein contacts and consequently the probability of protein crystallization. Several alternatives have been proposed such as amphipols, large polymers that efficiently stabilize membrane proteins by surrounding their lipophilic domain but remain poor detergents [7], [8]. Peptide-like detergents such as lipopeptides have also been developed to mimic membrane domains [9]. Maltoside cholane derivatives have been conceived to cover large stretches of membrane domains [10]. More recently maltose–neopentyl glycol (MNG) amphiphiles have been designed to stabilize integral membrane proteins [11]. All these solutions, although elegant, yet have not significantly opened up the field. In the present work, we have designed a new generation of detergents. They have been conceived for increasing the stability of the extracted/purified membrane protein by maintaining a higher rigidity of the membrane domain than that obtained by classical detergents, and closer to that imposed by lipids in a bilayer membrane environment. We have demonstrated the utility of these compounds by extracting different membrane proteins from prokaryote to higher eukaryote cell lines. Moreover, these compounds increased the stability of one membrane protein, BmrA, which was demonstrated to be maintained in its active state during extraction and purification, even after detergent exchange, and finally promoting its 3D crystallization.

## Materials and Methods

### C4Cn stock solution preparation

Compounds were prepared at 10 mg/ml in water, buffered to pH 8.0 (or 6 to 9 when indicated) with NaOH/HCl, the final salt concentration being adjusted to 0.3 M.

### Surface tension measurements

Surface tension properties of C4Cn were determined on a 1-ml drop using a Kibron µ-Trough and with a stainless steel needle in the Wilhemy balance. Curves were fitted with Sigmaplot v11. CMC values were calculated from the resulting plots as the intersection between the plateau reached at the minimal value of the surface tension and the tangent of each curve.

### Dynamic light scattering (DLS)

C4Cn suspensions were diluted to 10 mM, sonicated for 1 min (10 on/off cycles of 3 sec) and centrifuged for 1 h at 100,000 g prior to determination of the apparent dynamic radius on a Zetasizer NanoS apparatus (Malvern), measurements were repeated six times. The mean size distribution was generated as an intensity distribution then converted to a volume distribution and a number distribution using the DTS software v5.0.

### NMR

^1^H NMR spectra of 10 mM of the respective amino acids (99%+, ACROS) prepared in D_2_O were recorded on a Bruker Avance II-200 (500.13 MHz for ^1^H) spectrometer equipped with a BBO BB-1H/D Z-GRD direct broad-band probe. C4C1 was prepared as above, but with NaOD, DCl and D_2_O. ^1^H NMR titration experiments were carried out by varying the amino acid to C4C1 ratio from 0.1 to 20.

### Protein expressions and membrane preparations

ABC transporter enriched-membranes were prepared as described, for BmrA [12] and YheI/YheH (a *Bacillus subtilis* ABC transporter) [13] expressed in *E. coli*, whereas BCRP was expressed in either *Spodoptera frugiperda* (*Sf*9) insect cells [14] or human embryonic kidney (HEK293) cells [15]. Membrane fraction from HEK293 cells was prepared as follows: cells were incubated for 1 h in a hypotonic buffer containing 10 mM Tris-HCl pH 8.0, 10 mM KCl, 5 mM EDTA and supplemented with protease inhibitors (Sigma). Crude membranes were prepared by homogenizing cells with a Potter-Elvehjem homogenizer (Fisher Scientific Bioblock). Unbroken cells were removed by centrifugation at 1000 g for 15 min at 4°C. The supernatant was then centrifuged for 10 min at 10,000 g at 4°C, and then for 1 h at 100,000 g, at 4°C to pellet membranes. Membrane fractions were suspended at about 20 mg/ml before storage at −80°C in either 20 mM Tris-HCl, 1 mM EDTA, 0.3 M sucrose for BmrA or YheI/YheH, and 10 mM Hepes pH 8.0, 50 mM NaCl, 20% glycerol, 0.1 mM DTT, 1 mM EDTA for BCRP. Membrane fractions were analyzed on 10% SDS-PAGE (SDS polyacrylamide gel electrophoresis) [16] and their protein content estimated by the bicinchoninic acid method [17].

### Extraction assays

Membranes were thawed, and solubilisation was carried out for either 2 h (BmrA, BCRP) or 3 h (YheI/YheH) at 4°C with a protein concentration of 2 mg/ml (BmrA, BCRP) or 1 mg/ml (YheI/YheH). The C4Cn: protein ratio (w/w) was 5 (BmrA, BCRP) or 10 (YheI/YheH) in 20 mM Tris-HCl pH 8.0, 0.1 M NaCl (BmrA, BCRP) or 50 mM Tris-HCl pH 8.0, 15% glycerol, 100 mM NaCl, 10 mM imidazole, 5 mM β-mercaptoethanol (YheI/YheH). When indicated, solubilisations were carried out with DDM (Affimetrix) or FC12 (Affimetrix). Extracted and non-extracted materials were then separated by centrifugation at 100,000 g for 1 h. BCRP was detected by Western-blot using the BXP21 antibody (mouse, Millipore).

### ATPase activity

The ATPase activity was measured by quantifying the inorganic phosphate generated by ATP hydrolysis. Samples were incubated 20 min at 37°C in 50 mM Tris-HCl pH 8.0, 50 mM NaCl, 5 mM ATP, 5 mM MgCl_2_, and 2 mM NaN_3_. Experiments were carried out in the presence or absence of 1 mM orthovanadate to deduce the vanadate sensitive ATPase activity for ABC transporters [18]. The reaction was stopped by addition of 10% SDS before measuring the inorganic phosphate released [19].

### Purification of ABC transporters with C4Cn

The MSSSHHHHHH sequence was introduced by PCR at the 5′-end of the gene coding for BmrA, and cloned into the expression plasmid pET15b (Novagen). The protein (H_6_BmrA) was expressed in C41-DE3 *E. coli* strain and membrane fractions prepared as described [20]. Five milligram (proteins) of BmrA-enriched membranes were diluted to 2 mg/ml in 20 mM Tris-HCl pH 8.0, 0.5 M NaCl, protease inhibitors cocktail (Roche) and 10 mg/ml C4C3 were added, followed by an incubation of 2 h at 4°C and by centrifugation (100,000 g for 1 h at 4°C). C4C7 was added to the resulting supernatant at a concentration of 5 mg/ml, and the solution was subjected to the same treatment as mentioned above. The C4C7 supernatant was then analysed by DLS, as described above, or used for affinity and gel filtration chromatographies as described below. The C4C7 supernatant was bound to a nickel-nitriloacetic acid (NTA)-agarose resin of 1 ml (High Trap chelating hp GE Healthcare), then washed with 15 ml of 20 mM Tris-HCl pH 8.0, 0.5 M NaCl and 1 mg/ml C4C7. H_6_BmrA was eluted with a gradient from 0 to 100% of 20 mM Tris-HCl pH 8.0, 0.5 M NaCl, 250 mM imidazole and 1 mg/ml C4C7. The elution fractions were pooled, concentrated to 0.5 ml on Amicon Ultra4 50,000 (Millipore), loaded onto a Superdex 200 10/300 GL gel filtration resin (GE Healthcare) and eluted with 20 mM Tris-HCl pH 8.0, 0.1 M NaCl, 1 mg/ml FC12 as mobile phase.

### Fluorescence spectroscopy

Experiments were performed at room temperature using a Photon Technology International Quanta Master I spectrofluorimeter. All measurements were recorded in 500-µL quartz cuvettes (Hellma) with a 0.5-cm path length. Data were corrected for buffer contribution to fluorescence and the inner-filter effects of tested compounds by recording similar spectra with N-acetyl tryptophanamide as a control (NATA). BmrA (1 µM) (solubilized in FC12 or C4C10 and then purified with FC12) was in 20 mM Tris-HCl pH 8.0, 50 mM NaCl, 3 mg/ml FC12, 0.02 mM EDTA, 0.1 mM DTT and protease inhibitor cocktail. The fluorescence excitation was set at 295 nm, and the fluorescence emission scanned from 310 to 370 nm. The concentration-dependent quenching studies for daunorubicin (0–40 µM) and C4C10 (0–25 µM) were performed three times. Peak area of corrected fluorescence spectra were fitted with the SigmaPlot software (v11, Systat Software Inc.). Percentages of fluorescence quenching were calculated as a protein/NATA ratio. Fitting was achieved using the equation f = Fmin+((Fmax-Fmin)*((E+x+Kd)-SQRT((E+x+Kd)∧2-4*E*x)))/(2*E) [21], [22].

### Crystallization and x-ray diffraction data collection

BmrA crystallization assays were carried out using the sitting drop vapor diffusion method mixing equal volumes of protein and reservoir solution. Prior to flash freezing, crystals were transferred for 10–20 s to a 10 µl drop of reservoir solution containing 10% (v/v) glycerol for cryo-protection. Data were collected on beamline ID23EH-1 at the European Synchrotron Radiation Facility (ESRF, Grenoble).

## Results

### Concept and design of the structuring detergents

**Figure 1:**
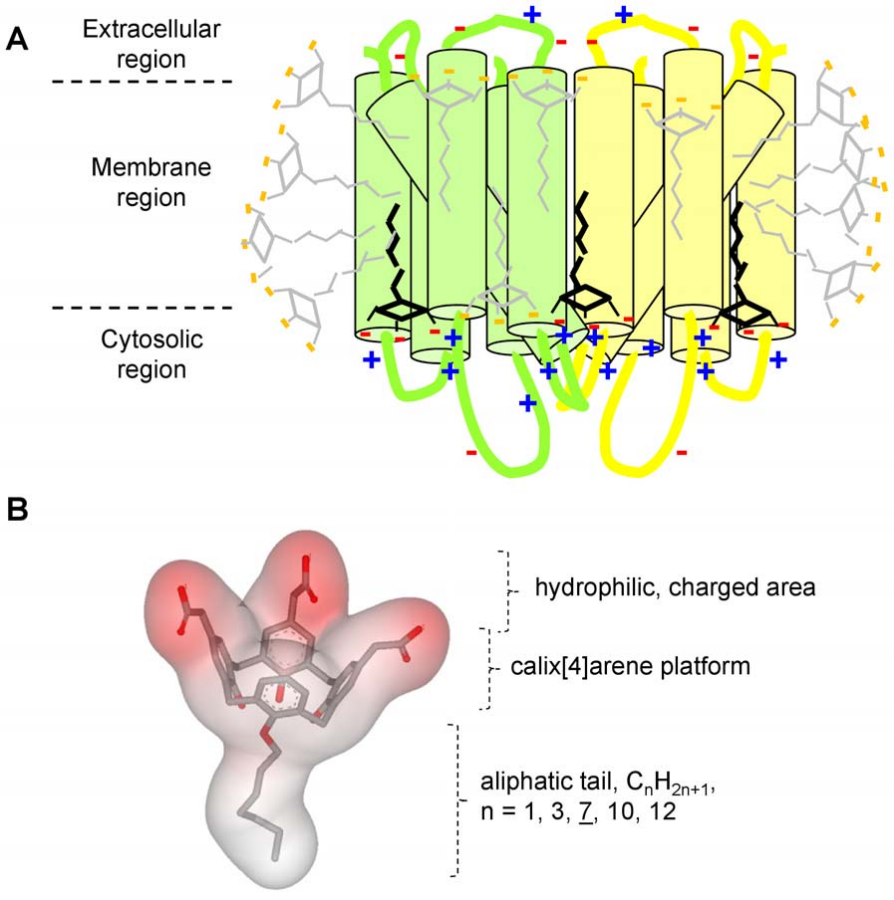
Concept of salt bridge network between anionic and amphiphilic molecules and basic residues located at the cytosol-membrane interface of membrane proteins. (A) *Scheme of a hypothetical dimeric membrane protein typically displaying basic residues at the cytosol-membrane interface, as established by von Heijne* [24]. In the absence of lipids, the membrane domain remains in a native conformation due to compounds displaying detergent properties for keeping the membrane protein in solution (grey molecules), but also mild-anionic groups (black molecules), for generating a network of salt bridges close to the membrane domain with basic amino acids carried by the intracellular loops (or domains) of the membrane proteins. (B) *Chemical structure of the designed molecules, C4Cn*. Three aromatic rings are substituted by a methylene carboxyl group, -CH_2_COOH, at the *para* position. An aliphatic chain R, O(CH_2_)_0-11_CH_3_, is grafted onto the fourth phenolic group. The resulting 3D-structure is modelled from the crystal structure of nitrile derivatives [26], substituting the CN groups with carboxyl groups.

In the late 80 s, Gunnar von Heijne established that membrane proteins from prokaryotes to higher eukaryotes relatively display a higher level of basic residues at the cytosol-membrane interface [23], [24], as schematized in Figure 1A. Originally validated on a limited set of membrane proteins, this observation was later strengthened by examining membrane proteins of more than one hundred genomes [25]. We took advantage of this feature to develop amphiphilic compounds able to build a network of salt bridges with these basic residues, in close proximity to the membrane region where such molecules accumulate upon membrane protein extraction. In addition to the favourable hydrophobic interactions between the hydrophobic chains of the detergent molecules and the hydrophobic residues of the membrane domain, this network of salt bridges is expected to strengthen the membrane-domain packing. To test this concept, we conceived a set of amphiphilic calix[4]arene-based constructs for which we previously reported the synthesis [26]. These compounds are built on a rigid calix[4]arene backbone onto which three acidic methylene-carboxylate groups have been grafted at the *para* position while the other face bears a single aliphatic chain C_n_H_2n+1_ varying in length from one to twelve carbon atoms (Figure 1B). The latter allows the modulation of the hydrophobic properties of the molecule to optimize the step of membrane protein extraction. The overall constructs possess a more or less elongated tetrahedral shape with three negatively-charged summits and one hydrophobic tail, which should favour the formation of spherical micelles [27]. They are called “C4Cn” in the present paper, with n = 1–12.

### Biophysical properties of C4Cn and interaction with basic amino acids

**Figure 2:**
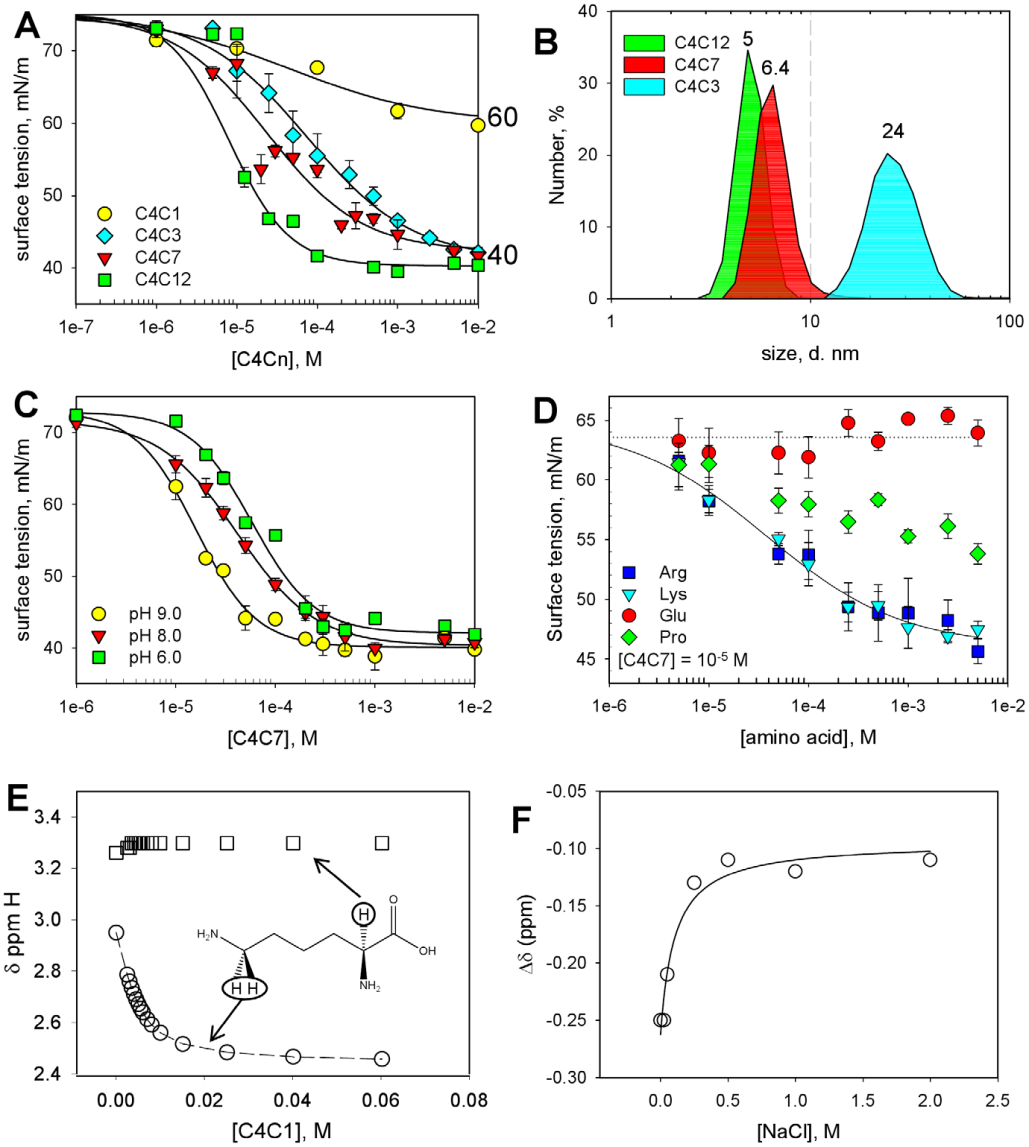
C4Cn behaviour in solution and interaction with basic amino acids. (A) *Effects of increasing C4C1, C4C3, C4C7 and C4C12 concentrations on the surface tension of aqueous solution*. The surface tension is measured as described in Methods. Each value is the mean of three experiments ± the standard error. C4C1, C4C3, C4C7 and C4C12 are indicated by circles, diamonds, triangles and squares, respectively. (B) *Dynamic light scattering of C4Cn in aqueous solution*. Experiments were carried out as described in Methods. Mean size values are indicated in nm for the corresponding compound. (C) *Effect of pH on the surface tension generated by C4C7*. Experiments have been carried out as in (A), neutralizing C4C7 at pH 9.0, 8.0 and 6.0, and measuring the resulting surface tension by increasing the concentration of the compound, as indicated by circles, triangles and squares, respectively. The values result from triplicate experiments. (D) *Interaction of C4C7 with amino acids probed by surface tension*. C4C7 diluted to 10 µM was incubated with increasing concentrations of either glutamate (circles), lysine (triangles), arginine (squares) or proline (diamonds), measuring the resulting surface tension, as described in Methods. (E) *Chemical shifts of the αH and εH protons of lysine in the presence of C4C1*. The ^1^H NMR spectra of L-Lysine (10 mM) was recorded as described in Methods in the presence of increasing C4C1 concentrations as indicated in the Figure, resulting in chemical shifts of αH and εH protons of lysine which were plotted as a function of C4C1 concentration. (F). *Dissociation of the L-lysine - C4C1 complex*, each added at 10 mM, induced by increasing the salt concentration as indicated and probed by measuring the chemical shift of lysine αH protons.

C4Cn form colloidal suspensions at pH values above 5.0, and can be used in a pH range extending from 5.5 to 14. They display a wide range of CMC values varying from 1.5 mM for C4C3 to 0.05 mM for C4C12, with a clear but surprising rupture in the CMC values at C4C7 (Figure 2A). As estimated from dynamic light scattering (DLS), the micelles of C4Cn have a mean-size diameter of 5–24 nm (Fig. 2B). These molecules being weak acids, their CMC is sensitive to the pH, as exemplified for C4C7 in Figure 2C, which shows that lowering the pH from 9 to 6 increases the CMC from 0.07 mM to 0.3 mM. Such a property can be used for modulating the micellar behaviour of these detergents. We then followed the interaction of C4Cn with amino acids, again illustrated with C4C7, probed as a variation of the surface tension. As shown in Figure 2D, increasing the concentration of an anionic amino acid such as glutamatic acid (circles) had no effect; the effect of proline (diamonds) remained limited while that of basic amino acids such as arginine (squares) or lysine (triangles) markedly lowered the surface tension. This behaviour was confirmed by ^1^H NMR titration experiments [28] carried out with C4C1 and lysine (Figure 2E), which clearly shows that the signals for the εCH_2_ protons of lysine are strongly shifted to higher field upon interaction with calix[4]arenes while the αH signal remains constant. The inclusion is stabilised by the formation of a salt bridge between the terminal NH_3_^+^ and a carboxylate and can be reversed by increasing the salt concentration above 0.5 M (Figure 2F).

### C4Cn successfully extract membrane proteins from prokaryotes to higher eukaryotes

**Figure 3:**
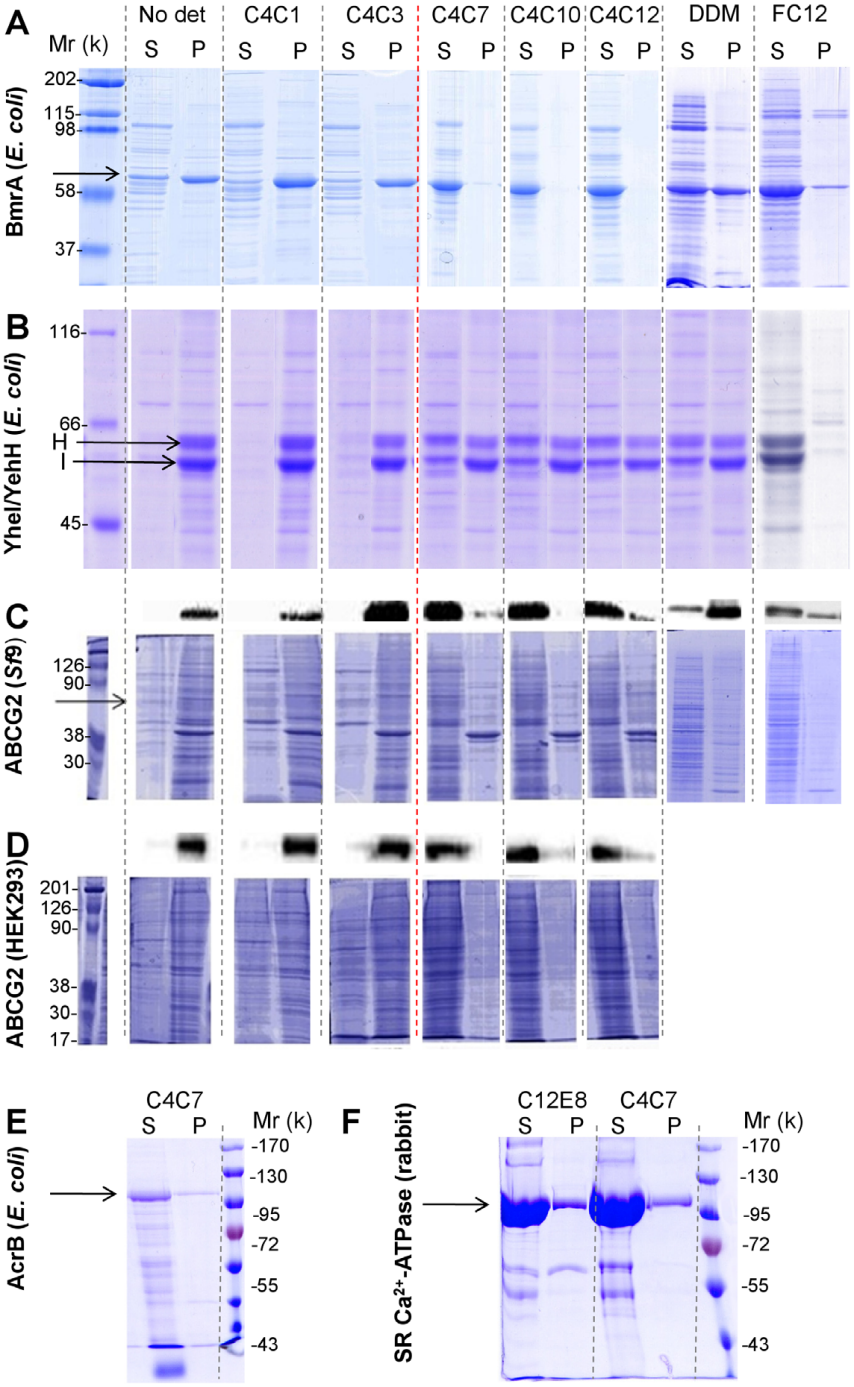
Extraction of membrane proteins by C4Cn derivatives. Extraction of ABC transporters by C4Cn, prokaryotic BmrA (A) and YheI/YheH (B) expressed in *E. coli*, human ABCG2 expressed in *Sf*9 (C) or HEK293 (D) cells, together with AcrB expressed in *E. coli* (E) and the SR-Ca^2+^-ATPase (F), was carried out on the corresponding membrane fractions as described in Methods. After solubilisation, extracted and non-extracted materials were separated by high-speed centrifugation, generating a supernatant S and a pellet P which were loaded on a 10% SDS-PAGE, and after migration either stained with Coomassie blue or submitted to a Western blot for ABCG2 (upper lanes in panels in C and D). Arrows indicate the position of each monomer. Positive control experiments were carried out with DDM, FC12 and C12E8, negative controls were carried out without detergent (*No det*). The red dotted line indicates the threshold of extraction.

The ability of the new tri-anionic calix[4]arene-based detergents to extract membrane proteins was demonstrated for a number of different membrane proteins: two ABC transporters from *Bacillus subtilis* expressed in *E. coli*, the homodimer BmrA [12] and the heterodimer YheI/YheH [13], the human homodimeric ABC transporter ABCG2 [29], [30] expressed either in *Sf*9 insect cells [14] or in human embryonic kidney HEK293 cells [15]. We also tested membrane proteins being unrelated to ABC transporters such as AcrB overexpressed in *E. coli* [31] and the naturally abundant sarcoplasmic reticulum calcium-transporting ATPase [32]. As shown in Figure 3A–D, compounds bearing a short aliphatic tail such as C4C1 and C4C3 did not extract the membrane proteins but contributed to remove contaminating proteins in the corresponding supernatants. A break in the extraction properties of the compounds was clearly observed from C4C7 and above, as was seen in the physical properties of the molecules. C4C12 extracted BmrA (panel A) or ABCG2 expressed in insect cells (panel C) with a higher efficacy than DDM and at least as well as FC12. As shown in panel B, C4C7, C4C10 and C4C12 extracted only 30–50% of the YheI/YheH heterodimer. A similar result was obtained with DDM, while FC12 (or SDS, not shown) fully extracted the heterodimer from the membrane fraction. This arises from the fact that this heterodimer is probably non-homogeneously overproduced in *E. coli*, one population of the protein being extracted only by strong detergents. These results also highlight that, although anionic, these compounds behave more like mild detergents, such as DDM, than as more aggressive detergents such as FC12 (or even stronger ones such as SDS). Consequently, they will extract membrane proteins under mild conditions but will likely fail to solubilize proteins from objects such as inclusion bodies. Finally, although mainly exemplified with ABC transporters, the extraction capacity of C4C7-12 is not limited to these proteins as shown in panels E and F with C4C7 extracting AcrB and the SR-Ca^2+^-ATPase, respectively (E and F panels).

### C4Cn allow BmrA purification and detergent exchange

**Figure 4:**
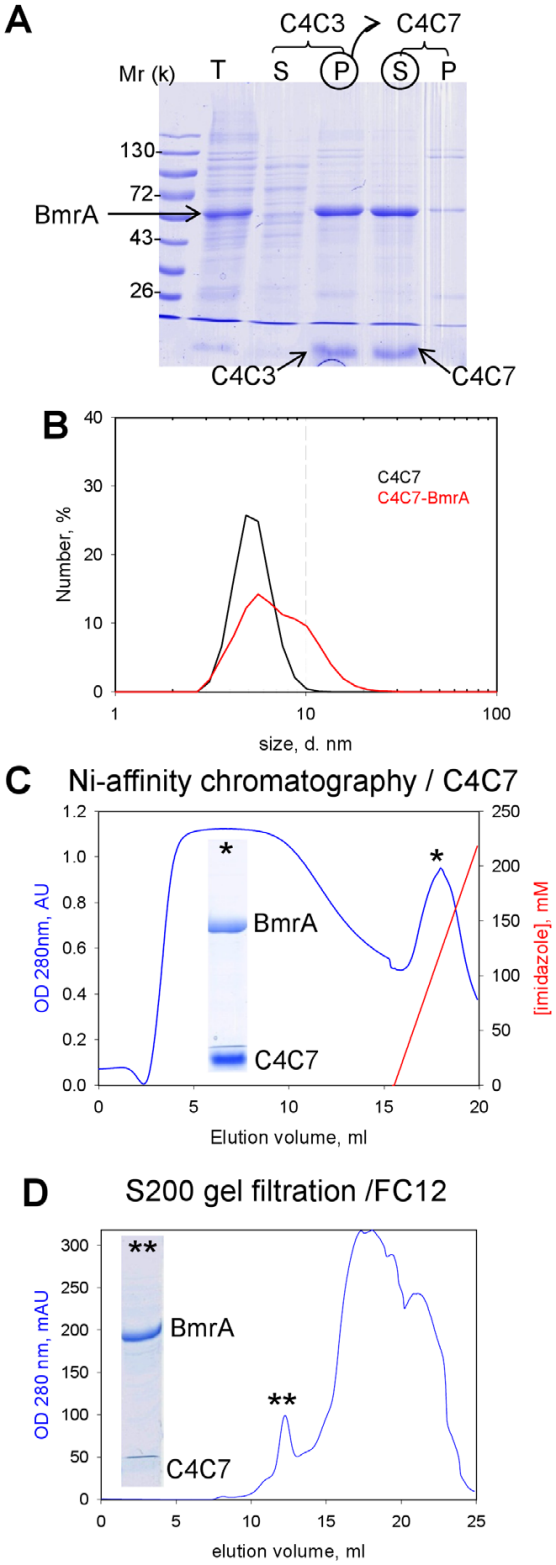
BmrA purification with C4Cn and detergent exchange. (**A**) SDS-PAGE of the sequential extraction of BmrA. As detailed in Methods, the membrane fraction (lane T) was incubated with C4C3 and then centrifuged to give the supernatant S and the pellet P. The latter, enriched in BmrA, was suspended in the presence of C4C7 and then centrifuged to give the corresponding supernatant S and pellet P. Arrows indicate the position of BmrA, C4C3 and C4C7. The C4C7 supernatant was then subjected to DLS (B), Ni-affinity chromatography (**C**) and gel filtration carried out with FC12 (**D**) from which respective pools indicated by stars were loaded onto SDS-PAGE.

Purification is a pre-requisite step and detergent exchange is routinely carried out for subsequent membrane protein functional studies and crystallization. The compounds were tested in such assays. Taking advantage of the poor capacity of C4C3 to extract BmrA (Fig. 3), we carried out a first step with that compound to discard contaminating proteins in the resulting supernatant (Fig. 4A, lane C4C3 S) and retain the desired protein in the precipitate. The BmrA-enriched membrane pellet obtained with C4C3 (lane C4C3 P) was then subjected to C4C7 extraction allowing total extraction of BmrA (lane C4C7 S). As shown, C4Cn could be visualized at the bottom of the SDS-PAGE (arrows), due to their interaction with the Coomassie blue stain. This is characteristic of the calix[n]arenes, classical detergents being not revealed by this stain. The C4C7 supernatant, mainly containing BmrA, displayed as shown using DLS (Fig. 4B), a population of mixed protein-detergent micelles of 10-nm diameter (red trace) in addition to that of C4C7 micelles centred to 5-nm diameter (black trace). The diameter of the latter is somewhat smaller than that estimated from Figure 2B (6.4 nm), a difference that can be attributed to the different buffer compositions. BmrA from the C4C7 supernatant was further purified by nickel-affinity chromatography carried out in the presence of C4C7 (see Methods for details), as shown in Figure 4C. The protein bound to the resin and eluted in a single peak (star) is displayed in the corresponding SDS-PAGE. The same C4C7 supernatant was alternatively submitted to gel filtration chromatography carried out in the presence of FC12 (or DDM, not shown) to allow the exchange of C4C7 by FC12. Results are displayed in Figure 4D. As shown, BmrA eluted at 12 ml (twin stars), an elution volume typical of a dimer, the C4C7 micelles eluting later between 15 and 25 ml. The detergent exchange could be easily checked using SDS-PAGE, which shows a faint trace of C4C7 at the bottom of the gel, by comparison with the SDS-PAGE done with the Ni-NTA pool (SDS-PAGE Fig. 4C).

### C4Cn preserve a functional state of BmrA during extraction, purification and detergent exchange

**Figure 5:**
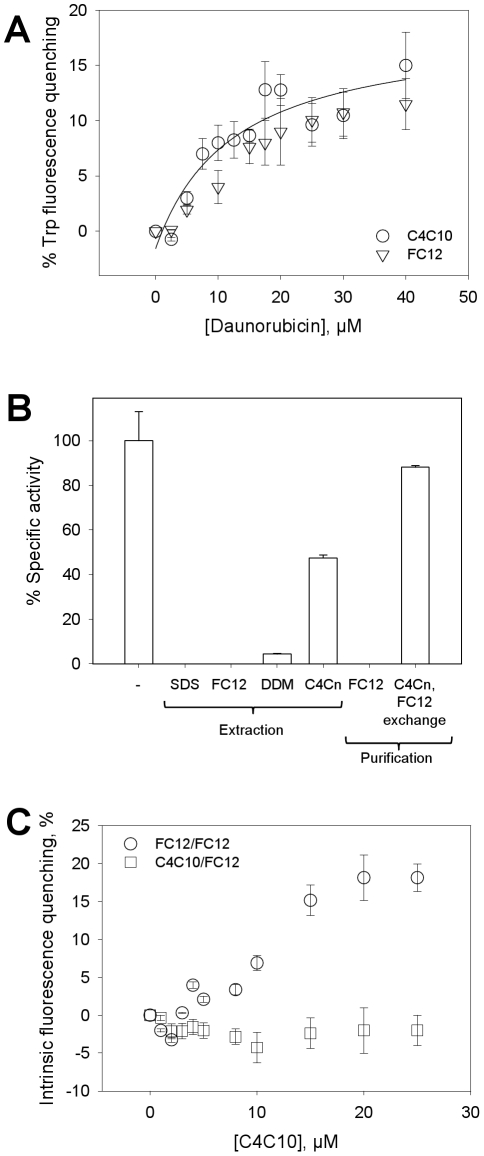
C4Cn preserve a functional state of BmrA. (**A**) Binding of daunorubicin to BmrA monitored by intrinsic (Tryptophan, Trp) fluorescence quenching. BmrA was extracted and purified either with C4C10 (circles) or FC12 (triangles) as in Figure 4, the former being subsequently exchanged by FC12 as in Figure 4D. The purified protein was then incubated with increasing concentrations of daunorubicin, the binding of the drug being probed by the variation of intrinsic fluorescence of BmrA, as described in Methods. (**B**) VO_4_-sensitive ATPase activity of BmrA (see Methods) in different fractions: BmrA-enriched membrane fraction (“-“ bar) corresponding to 0.5 µmol/min.mg and taken as 100%; BmrA-enriched membrane fraction solubilized with 1% SDS, FC12, DDM, or C4C3+C4C7 (“Extraction/C4Cn” bar, carried out as in Fig. 4); BmrA extracted with FC12 and then purified by metal affinity and gel filtration with FC12 (“Purification/FC12” bar); BmrA extracted with C4C3+C4C7 and then purified by metal affinity with C4C7 followed by detergent exchange with FC12 using gel filtration as carried out in Figure 4 (“Purification/C4Cn, FC12 exchange” bar). (**C**) Intrinsic fluorescence quenching monitoring of C4C10 binding on BmrA. BmrA was extracted either with C4C10 or FC12 and then purified with FC12 as in Figure 4 generating two populations on which C4C10 binding was monitored by probing the quenching of intrinsic fluorescence of 1 µM BmrA as detailed in Methods.

The aim of this work was to synthesize detergents able to maintain functional membrane proteins in detergent solution for further functional and crystallization assays. We checked the ability of the new compounds to achieve this task using BmrA. As for all ABC transporters, BmrA displays a membrane domain and a cytosolic domain, the former binding and transporting drugs and the later binding and hydrolysing ATP. Transport occurs through successive conformational changes involving a cross-talk between cytosolic and membrane domains. Hydrolysis of ATP allows resetting of the protein to its initial conformation after drug efflux [33], [34]. The protein in detergent solution cannot transport a drug due to the absence of closed vesicles but it should remain able to carry out all the steps of drug binding and release associated with ATP hydrolysis. This ATPase activity is therefore a useful tool for probing the functional state of ABC transporters. We used it together with the drug-binding capacity of the transporter to evaluate the effects of our compounds in comparison with the effects induced by commonly-used detergents such as DDM, FC12 and also SDS as an anionic detergent. The results are displayed in Figure 5. In a first experiment displayed in panel A, we compared the drug-binding capacity of BmrA extracted with either FC12 or C4C10. As C4C10 interferes with the fluorescence of tryptophan residues, it was necessary to exchange it with FC12 after Ni-NTA chromatography (done as shown in Figure 4). The addition of daunorubicin, a drug previously shown to be transported by BmrA [12], led to quenching of tryptophan fluorescence saturating at 40 µM with 15% quenching. As shown, the binding of daunorubicin was similar for both detergents. The resulting Kd could be estimated as 12.4±0.8 µM, a value statistically identical to that found for the protein previously purified in DDM (12.2±3.5 µM) [12]. This result supports the fact that the membrane domain of BmrA remains fully functional under these conditions.

Results were much more variable when we checked the ATPase activity of BmrA remaining after extraction with these detergents. As shown in Figure 5B the extraction by DDM, FC12 or SDS under conditions used in Figure 3 resulted in a reduction of 90–99% of the ATPase activity, indicating a loss of communication between membrane and cytosolic domains and suggesting that the protein was somehow altered and/or inhibited. In contrast, C4C7 used under the same conditions fully extracted BmrA and preserved up to 50% of the ATPase activity (Fig. 5B, Extraction/C4Cn bar). Finally, we checked the ATPase activity of the protein after extraction with C4C3+C4C7, followed by Ni-NTA chromatography in C4C7 and gel filtration carried out with FC12 (experimental conditions of Figure 4). The activity of this extract was compared to that of the protein purified using only FC12. Strikingly, we observed that in the first case the protein recovered 90% of ATPase activity although FC12 was present in the last step of purification whereas when extracted and purified with just FC12 no ATPase activity was detected. This suggests that C4C7 remaining associated to BmrA after exchange with FC12, (see SDS-PAGE in Figure 4D), may be sufficient to maintain BmrA in a functional state. It should be noted that these ATPase activities were measured 48 h after purification, using fractions stored at 4°C, indicating that the stabilisation obtained with C4C7 is not transient.

The functional stabilisation brought about by C4Cn supposes that this kind of detergent through hydrophobic interactions and multiple salt bridges should have a stronger degree of interaction with the membrane as compared to the interactions with classical detergents. To investigate this point, we probed the interaction of C4C10 with BmrA, extracted either with C4C10 or FC12 and then purified in FC12 by following the intrinsic fluorescence of the membrane protein. As shown in Figure 5C, the addition of C4C10 to the protein extracted with FC12 and purified with FC12 (circles) led to a quenching of about 20%, obtained at a concentration of 20 µM of detergent. This corresponds to a detergent-protein ratio of 20, BmrA being at a concentration of 1 µM in this experiment (see legend of the Figure). In contrast, no quenching of fluorescence was observed for the protein extracted with C4C10 and purified with FC12 (squares) suggesting that here the binding sites of C4C10 were already saturated by this detergent during extraction and that it remained bound to the protein during the subsequent FC12-exchange step. This implies that C4C10 interacts specifically with the membrane domain which could explain the functional stability of the protein in this detergent solution.

### C4Cn promote BmrA crystallization

Anionic detergents have been reported to not favour membrane protein crystallization. Because C4Cn belong to this class, we investigated the consequences of their use in this type of assays. In contrast to certain ABC transporters [35], [36], [37], BmrA has not been yet structurally characterised and none of them have been structurally characterised in the presence of drugs. We are thus trying to elucidate the 3D structure of BmrA in the presence of drugs, and towards this end the action of C4C10 and FC12 with regard to crystallization has been compared.

**Figure 6:**
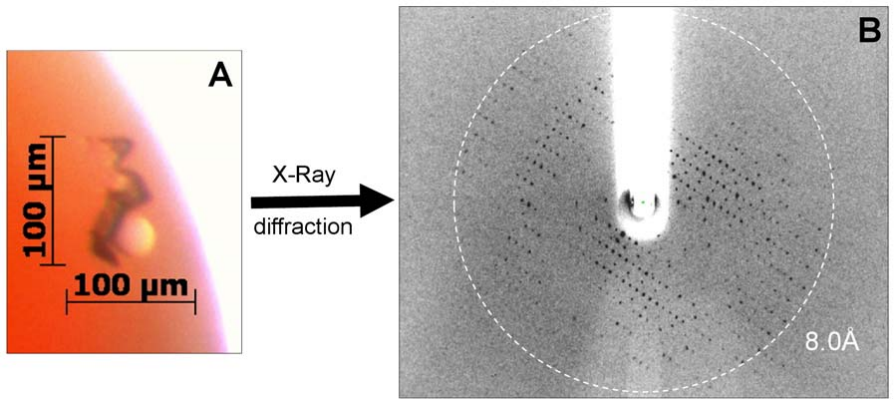
Crystallization of BmrA extracted with C4C10. BmrA was extracted and purified as described in Figure 5, using C4C10 instead of C4C7 and exchanging it with FC12. (A) The protein, concentrated to 10 mg/ml, and mixed with 1 mM doxorubicin, crystallized after 10 days in 0.2 M KSCN, 20% PEG 3350, and was (B) analyzed at the ESRF beamline ID23EH-2.

BmrA was extracted either with C4C10 followed by exchange with FC12 as described above or directly extracted and purified with FC12. Crystals were obtained under several conditions using the C4C10-extracted protein, including 0.2 M KSCN, 20% PEG 3350 and 1 mM doxorubicin as displayed in Figure 6A. However, no crystals were observed under the same conditions with BmrA that was directly extracted and purified in FC12. This illustrates that although anionic, C4Cn detergents may promote membrane protein crystallization. It also suggests a key role of the tightly bound C4C10 for promoting BmrA crystallization. This hypothesis is supported by the fact that the crystals displayed in Fig 6A diffracted X-rays to 8.0 Å resolution (Figure 6B).

## Discussion

Structural biology of membrane proteins is still in its infancy, being constrained by two hardly-compatible principles: the need of lipids for structuring the membrane domain, and the imperfect folding of proteins outside this specific environment. However, the techniques used to date to solve their 3D-structure require detergent-solubilized membrane proteins, except for the promising approach of solid-state NMR, still in an early development stage [38] and which cannot be used (to date) for solving the structure of membrane proteins as large as BmrA (130 kDa). Amphiphilic molecules such as detergents are thus used to make the link between these constraints, replacing lipids around the membrane through hydrophobic interactions and maintaining the protein in micelles through hydrophilic interactions with the solvent. Unfortunately, they display poor structuring properties. The current compounds were designed to fill this gap, adding to these classical amphiphilic properties the capacity to generate a network of salt bridges around the protein, in close proximity to the membrane domain with positively-charged residues located at the membrane-cytosol interface of the protein [24]. Figure 2 shows that the compounds reported herein indeed are amphiphilic molecules, generating micelles (panel A) and, as expected interact with positively-charged amino-acids (panel D). Although bearing three negative charges, these detergents do not behave as FC12 or SDS. Heptyl to dodecyl derivatives of C4Cn extract membrane proteins tested here (Figure 3), in some cases partially (YheI/YheH), behaving like mild detergents, as shown here with BmrA, ABCG2, AcrB and the Ca^2+^-transporting ATPase. An advantage of the present set of compounds lies in the fact that the derivatives carrying short alkyl chains can be used to enrich the fraction containing the target protein, as shown in Figure 3 for all the tested ABC transporters and also, more specifically, for BmrA in Figure 4A. This behaviour allows using a limited number of trials, optimization of the extraction step, thus avoiding the necessity to test a large number of assays as needed for other detergents.

We have designed these detergents with the aim of stabilizing an active conformation of the extracted membrane proteins such as ABC transporters, and as shown in Figure 5 they indeed behave as expected. As exemplified by C4C7 and C4C10, this new type of detergents preserves the ATPase activity together with a drug-binding capacity to the extracted protein. Both the extraction and the retention of activity are better than those observed with mild detergents such as DDM, zwitterionic and more aggressive detergents such as FC12, or anionic and stronger detergents such as SDS. Although rather encouraging, the decrease of 50% of ATPase activity upon C4Cn extraction shows that these compounds can be improved. Quite importantly, they can be used throughout a purification process, including metal affinity and gel filtration chromatographies, a last step allowing if required a successful exchange with a classical detergent such as FC12 (Figure 4D), with a preservation of the protein in an active state while the same process carried out with FC12 results in an inactive protein. This may be due to a tight binding of C4Cn as suggested by Fig. 5C which shows that C4Cn interact with BmrA when extracted and remains bound to BmrA in the presence of FC12 when added in the last purification step. This small set of compounds thus represents a powerful tool for the study of ABC transporters but also for the extraction of other membrane proteins, *e.g*. AcrB or SR Ca^2+^-ATPase, as shown (Fig. 3). Last but not the least, these new compounds, although anionic, seem to be potentially promising in promoting the crystallization of membrane proteins as seen here for BmrA. Further work will aim at improving the quality of the crystals so as to allow determination of the 3D-structure of this and other membrane proteins, hopefully at a resolution high enough to identify C4Cn bound to the protein.

## References

[1] Wallin E, Von-Heijne G (1998). Genome-wide analysis of integral membrane proteins from eubacterial, archaean, and eukaryotic organisms.. Protein Sci.

[2] Overington JP, Al-Lazikani B, Hopkins AL (2006). How many drug targets are there?. Nat Rev Drug Discov.

[3] White SH (2009). Membrane Proteins of known 3D structure.. http://blanco.biomol.uci.edu/Membrane_Proteins_xtal.html.

[4] Seddon AM, Curnow P, Booth PJ (2004). Membrane proteins, lipids and detergents: not just a soap opera.. Biochim Biophys Acta.

[5] van Meer G, Voelker DR, Feigenson GW (2008). Membrane lipids: where they are and how they behave.. Nat Rev Mol Cell Biol.

[6] Matthews EE, Zoonens M, Engelman DM (2006). Dynamic Helix Interactions in Transmembrane Signaling..

[7] Tribet C, Audebert R, Popot JL (1996). Amphipols: polymers that keep membrane proteins soluble in aqueous solutions.. Proc Natl Acad Sci U S A.

[8] Champeil P, Menguy T, Tribet C, Popot JL, le Maire M (2000). Interaction of amphipols with sarcoplasmic reticulum Ca^2+^-ATPase.. J Biol Chem.

[9] McGregor CL, Chen L, Pomroy NC, Hwang P, Go S (2003). Lipopeptide detergents designed for the structural study of membrane proteins.. Nat Biotechnol.

[10] Zhang Q, Ma X, Ward A, Hong W-X, Jaakola V-P (2007). Designing Facial Amphiphiles for the Stabilization of Integral Membrane Proteins.. Angewandte Chemie International Edition.

[11] Chae PS, Rasmussen SGF, Rana RR, Gotfryd K, Chandra R (2010). Maltose-neopentyl glycol (MNG) amphiphiles for solubilization, stabilization and crystallization of membrane proteins..

[12] Steinfels E, Orelle C, Fantino JR, Dalmas O, Rigaud JL (2004). Characterization of YvcC (BmrA), a multidrug ABC transporter constitutively expressed in Bacillus subtilis.. Biochemistry.

[13] Torres C, Galián C, Freiberg C, Fantino J-R, Jault J-M (2009). The YheI/YheH heterodimer from Bacillus subtilis is a multidrug ABC transporter.. Biochimica et Biophysica Acta (BBA) - Biomembranes.

[14] Trometer C, Falson P, Mus-Veteau I (2010). Mammalian membrane proteins expression in baculovirus infected insect cells.. Heterologous expression of membrane proteins: methods and protocols. 2010 ed:.

[15] Boumendjel A, Macalou S, Ahmed-Belkacem A, Blanc M, Di Pietro A (2007). Acridone derivatives: Design, synthesis, and inhibition of breast cancer resistance protein ABCG2.. Bioorg Med Chem.

[16] Montigny C, Penin F, Lethias C, Falson P (2004). Overcoming the toxicity of membrane peptide expression in bacteria by upstream insertion of Asp-Pro sequence.. Biochim Biophys Acta.

[17] Smith PK, Krohn RI, Hermanson GT, Mallia AK, Gartner FH (1985). Measurement of protein using bicinchoninic acid.. Anal Biochem.

[18] Hamada H, Tsuruo T (1988). Characterization of the ATPase Activity of the Mr 170,000 to 180,000 Membrane Glycoprotein (P-Glycoprotein) Associated with Multidrug Resistance in K562/ADM Cells.. Cancer Res.

[19] Drueckes P, Schinzel R, Palm D (1995). Photometric microtiter assay of inorganic phosphate in the presence of acid-labile organic phosphates.. Anal Biochem.

[20] Chami M, Steinfels E, Orelle C, Jault JM, Di Pietro A (2002). Three-dimensional structure by cryo-electron microscopy of YvcC, an homodimeric ATP-binding cassette transporter from Bacillus subtilis.. J Mol Biol.

[21] Falson P, Penin F, Divita G, Lavergne JP, Di Pietro A (1993). Functional nucleotide-binding domain in the F0F1-ATPsynthase alpha subunit from the yeast *Schizosaccharomyces pombe*.. Biochemistry.

[22] Desuzinges-Mandon E, Arnaud O, Martinez L, Huché F, Di Pietro A (2010). ABCG2 Transports and Transfers Heme to Albumin through Its Large Extracellular Loop.. Journal of Biological Chemistry.

[23] von Heijne G, Gavel Y (1988). Topogenic signals in integral membrane proteins.. European Journal of Biochemistry.

[24] von Heijne G (1992). Membrane protein structure prediction. Hydrophobicity analysis and the positive-inside rule.. J Mol Biol.

[25] Nilsson J, Persson B, von Heijne G (2005). Comparative analysis of amino acid distributions in integral membrane proteins from 107 genomes.. Proteins: Structure, Function, and Bioinformatics.

[26] Suwinska K, Shkurenko O, Leydier A, Mbemba C, Jebors S (2008). Tri-Anionic Calix[4]arene Monoalkyl Derivatives: Synthesis, Solid-State Structures and Self-Assembly Properties.. New Journal of Chemistry.

[27] Israelachvili JN, Mitchell DJ, Ninham BW (1977). Theory of self-assembly of lipid bilayers and vesicles.. Biochimica et Biophysica Acta (BBA) - Biomembranes.

[28] Kalchenko O, Perret F, Morel-Desrosiers N, Coleman AW (2001). A comparative study of the determination of the stability constants of inclusion complexes of p-sulfonatocalix[4]arene with amino acids by RP-HPLC and ^1^H NMR.. Journal of the Chemical Society, Perkin Transactions.

[29] Ross DD, Yang W, Abruzzo LV, Dalton WS, Schneider E (1999). Atypical multidrug resistance: breast cancer resistance protein messenger RNA expression in mitoxantrone-selected cell lines.. J Natl Cancer Inst.

[30] Litman T, Brangi M, Hudson E, Fetsch P, Abati A (2000). The multidrug-resistant phenotype associated with overexpression of the new ABC half-transporter, MXR (ABCG2).. J Cell Sci.

[31] Deniaud A, Goulielmakis A, Covès J, Pebay-Peyroula E (2009). Differences between CusA and AcrB Crystallisation Highlighted by Protein Flexibility.. PLoS ONE.

[32] Lenoir G, Picard M, Moller JV, le Maire M, Champeil P (2004). Involvement of the L6-7 loop in SERCA1a Ca^2+^-ATPase activation by Ca^2+^ (or Sr^2+^) and ATP.. J Biol Chem.

[33] Higgins CF, Linton KJ (2004). The ATP switch model for ABC transporters.. Nat Struct Mol Biol.

[34] Eckford PDW, Sharom FJ (2009). ABC Efflux Pump-Based Resistance to Chemotherapy Drugs.. Chemical Reviews.

[35] Dawson RJ, Locher KP (2006). Structure of a bacterial multidrug ABC transporter.. Nature.

[36] Ward A, Reyes CL, Yu J, Roth CB, Chang G (2007). Flexibility in the ABC transporter MsbA: Alternating access with a twist.. Proceedings of the National Academy of Sciences.

[37] Aller SG, Yu J, Ward A, Weng Y, Chittaboina S (2009). Structure of P-Glycoprotein Reveals a Molecular Basis for Poly-Specific Drug Binding.. Science.

[38] McDermott A (2009). Structure and dynamics of membrane proteins by magic angle spinning solid-state NMR.. Annu Rev Biophys.

